# Influence of Clinical Markers of Dopaminergic Behaviors on Depressive Symptoms During Withdrawal in Cocaine Users

**DOI:** 10.3389/fpsyt.2021.775670

**Published:** 2021-11-22

**Authors:** Julien Cabé, Georges Brousse, Bruno Pereira, Nicolas Cabé, Emily Karsinti, El-Hadi Zerdazi, Romain Icick, Pierre M. Llorca, Vanessa Bloch, Florence Vorspan, Ingrid De Chazeron

**Affiliations:** ^1^Service d'addictologie et pathologies duelles, Centre Hospitalier Universitaire de Clermont-Ferrand, Clermont-Ferrand, France; ^2^Université Clermont Auvergne, CHU, CNRS, Clermont Auvergne INP, Institut Pascal, Clermont-Ferrand, France; ^3^Faculté de Médecine, Université Clermont Auvergne, Clermont-Ferrand, France; ^4^Direction de la Recherche Clinique et des Innovations, CHU Clermont-Ferrand, Clermont-Ferrand, France; ^5^Normandie University, UNICAEN, PSL Université de Paris, EPHE, INSERM, U1077, CHU de Caen, GIP Cyceron, Neuropsychologie et Imagerie de la Mémoire Humaine, Caen, France; ^6^Service d'Addictologie, Centre Hospitalier Universitaire de Caen, Caen, France; ^7^INSERM UMR-S 1144, Université de Paris, Optimisation Thérapeutique en Neuropsychopharmacologie, Paris, France; ^8^Département de Psychiatrie et de Médecine Addictologique, Hôpital Fernand Widal, Assistance Publique–Hôpitaux de Paris, Paris, France; ^9^Service de Psychiatrie B, Centre Hospitalier Universitaire de Clermont-Ferrand, Clermont-Ferrand, France; ^10^Faculté de Médecine, Université de Paris, Paris, France

**Keywords:** cocaine, withdrawal, dopamine, clinical markers, depressive symptoms

## Abstract

**Background:** During cocaine withdrawal, transient depressive symptoms that do not meet the criteria for depression, but promote relapse, are frequently observed. Their temporality could evoke a role of dopamine, especially since the underlying mechanism of these depressive symptoms is not well understood. We hypothesized that variation in the dopaminergic activity profile, modeled from clinical markers, could be implicated in the development of depressive symptoms during cocaine withdrawal.

**Methods:** We compared patients reporting depressive symptoms (RDS+) or not (RDS–) during cocaine withdrawal. We evaluated dopaminergic activity through indirect clinical markers based on the known dopaminergic behaviors. A combined criterion was constructed for hyper and hypo dopaminergic models according to the O'Brien method and illustrated by the Hedges' effect-size and forest-plot graph. A multidimensional factorial analysis was carried out to determine which parameters discriminate RDS+/RDS– patients.

**Results:** 313 patients were included, and 77% reported depressive symptoms during cocaine withdrawal. Hyperdopaminergic variables used to discriminate the two groups had a large overall effect size (−0.669) and included psychotic symptoms (−0.524), hallucinations (−0.548), and delusions (−0.528). The overall effect of the hypodopaminergic component was considerable (−0.604) with a large effect size for the severity of dependence (−0.616), withdrawal symptoms (−0.578), and anhedonia (−0.528). The combined model including hyperdopaminergic and hypodopaminergic components had the largest effect size (−0.785).

**Conclusion:** The dopaminergic activities profile, assessed by indirect clinical markers, seems to characterize patients with depressive symptoms very well during cocaine withdrawal. RDS+ patients reported moreover higher levels of psychotic symptoms and more severe cocaine use disorder than RDS–.

## Introduction

An estimated 20 million individuals worldwide used cocaine according to the world drug report 2021. This global level of cocaine use continues to increase every year (1), and its impact in terms of public health is major, particularly with regard to overdoses (2). The treatment of this addiction is complex, especially when it is associated with a psychiatric disorder, with a high relapse rate and a worse follow-up addiction severity (3, 4). Cocaine, as well as other psychostimulants, can also cause stroke and alterations in mood and cognition (5).

Relapsing patients are more likely to declare serious life-time psychiatric symptoms, including depressive symptoms (6). These patients rated their psychiatric problems as more severe and reported a greater need for treatment for these problems (6). Some studies suggested that depressive symptoms are specifically and significantly associated with an increased risk of relapse after treatment in substance users (7). These mood fluctuations are important because they are a pejorative prognostic factor in cocaine-dependent patients (8) and are associated with increased suicidal risk (9). Furthermore, patients who drop out early have more depressive symptoms than the later dropouts (10). It has been shown that worse depressive symptoms represent a significant predictor of worse medical severity at 12-months follow-up (4). Depressive symptoms seem to play a key role in the process of relapse and so have been chosen in our study as a discriminative factor.

A particular form of transient depressive symptoms is observed during cocaine withdrawal. It is often the subsyndromic form and does not correspond to the well-known timeline of the major depressive disorder. There are several other specificities: (1) these depressive symptoms disappear when taking cocaine, (2) treatment of depression in cocaine users with selective serotonin reuptake inhibitor (SSRI) seems to underperform (11, 12). The mechanism therefore seems different and raises the question of the involvement of dopamine in the emergence of depressive symptoms during cocaine withdrawal. This is especially relevant considering that dopamine plays a central role in the mechanism of cocaine, which works by blocking the dopamine transporter (DAT) and increasing the brain's dopamine level (13). This involvement of dopamine had already been suggested by Dackis and Gold (14).

A potential model of dopaminergic depression can be observed in Parkinson's disease, where symptoms are correlated with dopamine levels (15). In this disease, the hypodopaminergic behaviors results in depression, anxiety, apathy, anhedonia, cognitive dysfunctions, and sleep disorders (15, 16). In the particular case of Parkinson's disease, the hyperdopaminergic behaviors, which is induced by dopaminergic treatments in Parkinson's disease, is characterized by hallucinations, delusions, and compulsive behaviors, such as pathological gambling, hypersexuality, shopping, binge eating, and punding (17, 18). These “dopaminergic behaviors” could be used as indirect clinical markers of dopamine activity.

The psychoactive effects of cocaine are fairly well described today (19). Among these symptoms are those that may serve as indirect markers of dopaminergic activity. Cocaine use induces a brief “peak” of pleasure, lasting a few minutes, associated with subjective stimulating effects. Sometimes, there are also psychotic symptoms, especially during high consumption: hallucinations, delusions, or consumption associated behavior (20). This phase then quickly gives way to a withdrawal characterized by contrary symptoms, such as depressive symptoms, anhedonia, and anxiety (21). The intensity of depressive and psychotic symptoms seems to be related to the severity of addiction and the level of use (22).

Based on the Parkinson's disease model and what is clinically observed in cocaine users, we hypothesized that variation in dopamine activity is implicated in the development of depressive symptoms during cocaine withdrawal. Consumers may first experience hyperdopaminergic symptoms upon substance use (psychotic symptoms, or stereotypes), then hypodopaminergic symptoms upon substance withdrawal (depressive symptoms, apathy, and anxiety).

Our objective is to investigate whether patients reporting depressive symptoms during cocaine withdrawal (RDS+) have a different profile of clinical markers of dopaminergic behaviors from those who do not (RDS–).

## Methods

### Study Design

This study is an analysis of secondary data from a French multicenter retrospective study called Psychocoke (23). The sample consisted of 313 cocaine users who sought treatment in drug treatment centers in France.

Inclusion criteria were: ≥18 years-old, medical follow-up for a current cocaine use disorder, social security affiliation. Exclusion criteria were be under protective supervision, have blood test contraindication, and not speak or understand French.

### Research Instruments

#### Sociodemographic Data

Sociodemographic and clinical information [e.g., age, gender, marital status, educational and professional level, personal medical history (psychiatric or addictive)] were collected from the staff-administered questionnaires.

#### Depressive Symptoms

We investigated whether patients subjectively felt the presence of depressive symptoms during cocaine withdrawal. Depressive symptoms in cocaine users are often assessed by self-report measures (22, 24). Some studies found that a significant proportion of patients with depressive symptoms do not meet the criteria for characterized depression during diagnostic evaluation (24). Standardized assessment scales are often not usable in the acute phase of drug use or withdrawal. Therefore, we assessed this aspect by asking them if they had ever experienced depressive symptoms during cocaine withdrawal with the following proposition: “Presence of depressive elements during the descent: No or Yes.” The group that reported depressive symptoms was named RDS+ (Reported Depressive Symptoms +), the other group was named RDS–.

#### Addiction Characteristics

Current and lifetime psychoactive substance use was evaluated in terms of consumption modality, age of onset, frequency, and amount of use for: cocaine, tobacco, opiates, alcohol, sedative drugs, amphetamines, ecstasy/MDMA, hallucinogens, ketamine, poppers, and cannabis.

The severity of cocaine dependence was assessed according to the criteria of DSM-IV (Diagnostical and Statistical Manual) (American Psychiatric Association, n.d.). We also wanted to estimate the severity of dependency with a dimensional approach, as is currently practiced with DSM 5. We therefore added the total number of DSM-IV criteria present for each patient, in order to be as close as possible to the current method of rating a substance use disorder (0 to 7/7 score).

#### Psychotic Symptoms

Cocaine-induced psychotic symptoms were assessed with the French version of the Scale for the Assessment of Psychotic Symptoms-Cocaine Induced Psychosis (SAPS-CIP) questionnaire (20, 25). This semi directive interview explores different dimensions: hallucinations (auditory hallucinations, visual hallucinations, somesthesic or tactile hallucinations, olfactory hallucinations), delusions (persecutory delusions, delusions of jealousy, delusions of sin or guilt, grandiose delusions, religious delusions, somatic delusions, ideas and delusions of reference, delusions of being controlled, delusions of mind reading), cocaine-associated behavior (aggressive and agitated behavior, repetitive or stereotyped behavior, social and sexual behavior, preparatory behavior) and physical symptoms prior to use (what the subject does to prepare for crack use: place of consumption, type of preparation, rituals, etc.). Each item was scored from 0 to 5, thus leading to a total score from 0 to 15.

### Procedures

Data collection was conducted from 2012 to 2016 through interviews performed by a trained psychologist or psychiatrist during a single visit.

### Data Analysis

Statistical analyses were performed using Stata software, version 13 (StataCorp, College Station, US) and R software with the ade4 package (http://www.R-project.org). The assumption of normality was checked using normal probability plots and the Shapiro-Wilk's test. The tests were two-sided, with a type I error set at 5%.

First, the comparisons between RDS–/RDS+ concerning categorical data were performed using the Chi-Squared test or Fisher's exact test, whereas the comparisons for quantitative variables among Reported Depressive Symptoms (no/yes) were analyzed using the Student *t* test or the Mann-Whitney test when the conditions of the *t* test were not met. Second, a combined criterion was constructed for hyper and hypo dopaminergic models according to the method developed by O'Brien (26). This framework allows the combination of multiple parameters into a single statistical assessment, without assigning a rank of relative importance.

Then, multidimensional factorial discriminant analysis (FDA) was carried out to uncover the underlying relationships parameters and to determine which parameters discriminated patients with and without RDS.

To illustrate these results and the magnitude of differences, Hedges' effect-size (i.e., difference of means between groups divided by the standard-deviation) and 95% confidence intervals were estimated and represented with a forest-plot graph.

### Ethical Aspects

The Research Ethics Committee of Ile de France (Paris area) approved the study protocol under Opinion NCT01569347. Written informed consent was obtained from all the participants.

### Results

Among the 313 cocaine-dependent subjects participating in the study, 77% (*N* = 241) showed depressive symptoms during the descent. The data presented in Table 1 show that there was no significant difference between the two groups for age (38.11 ± 9.28 vs. 38.32 ± 8.80), gender, marital status and school level. In our sample, we found mainly single men with a heterogeneous overall educational level (Table 1).

**Table 1:** Sociodemographic characteristics and psychotic symptoms (assessed by SAPS-CIP) of cocaine users with (RDS+) or without (RDS–) depressive symptoms during cocaine withdrawal.

**Variable**	**Without RDS (RDS–)**	**With RDS (RDS+)**	***p***
	**N = 72 (23%)**	**N = 241 (77%)**	
Age (years, mean ± SD)	38.11 (± 9.28)	38.32 (± 8.80)	0.87
Gender (Male, %)	77.78	78.84	0.85
Marital status (%)			
Single	84.72	75.52	
Married	6.94	13.69	0.22
Divorced	8.33	10.79	
School level (%)			
Primary	2.78	1.66	
Specialized	11.11	4.15	
Secondary 1st cycle	18.06	16.18	0.20
Secondary 2nd cycle	16.39	31.95	
Superior	41.67	46.06	
Psychotic symptoms (%)	45.83	70.12	<0.001^*^
Hallucinations score (mean ± SD)	1.00 (± 1.36)	1.81 (± 1.52)	<0.001^*^
Delusions score (mean ± SD)	1.60 (± 1.51)	2.35 (± 1.43)	<0.001^*^
Consumption-associated behavior score (mean ± SD)	1.85 (± 1.53)	2.20 (± 1.52)	0.09
Physical symptoms before use score (mean ± SD)	1.01 (± 1.41)	1.65 (± 1.45)	0.001^*^

* *Significant differences between groups according to independent samples t-tests or χ^2^ tests*.

A higher proportion of patients in the RDS+ group (70.12%) reported at least one experience of psychotic symptoms when using cocaine, compared to RDS- patients (45.83%) (*p* <0.001). With regard to detailed psychotic symptoms, there were significantly more hallucinations (*p* < 0.001), delusions (*p* < 0.001), and physical symptoms before use (*p* < 0.001) in the RDS+ group (Table 1).

All patients selected for the study had a lifetime of cocaine use and were current cocaine users (use within 1 month). The history of addiction hospitalization was similar in both groups and concerned nearly 65% of patients. The average age at which cocaine use began was similar in both groups (22.46 ± 7.06 vs. 23.31 ± 6.78 years), as well as the frequency of use (more than 65% of daily users) and the type of product consumed, mainly cocaine (Table 2). A significant difference in consumption patterns (*p* = 0.04) was observed. The nasal route of administration was more frequently found in the group of patients with depressive symptoms (63.90 vs. 48.61%). Injectable or smoked pathways were more frequent in the RDS+ group (26.39 vs. 23.65 % for smoked, 18.06 vs. 7.86 % for injectable). The consumption of other psychoactive substances (Tobacco, Opioid, Alcohol, Sedatives, Amphetamines, Ecstasy/MDMA, Hallucinogens, Ketamine, Poppers, Cannabis) was not significantly different between the two groups. The average number of substances used per patient did not differ.

**Table 2:** Clinical characteristics of cocaine users with (RDS+) or without (RDS–) depressive symptoms during cocaine withdrawal (*N* = 313).

**Variable**	**Without RDS (RDS–)**	**With RDS (RDS+)**	***p***
History of hospitalization for withdrawal (%)	66.67	64.73	0.76
Previous suicide attempt (%)	27.78	38.59	*0.09*
Age at first cocaine use (years, mean ± SD)	22.46 (± 7.06)	23.31 (± 6.78)	0.37
Frequency (Daily, %)	65.28	68.88	0.19
Cocaine use behavior (%)			
Crack	20.83	18.67	
Cocaïne	68.06	71.78	0.83
Crack + Cocaïne	11.11	9.54	
Administration			
Snorted	48.61	63.90	
Smoked	26.39	23.65	0.04^*^
Injected	18.06	7.88	
Injected + other way	6.94	4.56	
Poly-drug use in whole lifetime (number of substances, mean ± SD)	7.82 (1.89)	7.50 (1.94)	0.22
Cocaine dependency criteria (DSM IV, %)		
Tolerance	81.94	85.06	0.52
Withdrawal	61.11	84.23	<0.01^*^
Loss of control	91.67	90.04	0.82
Persistent desire	59.72	73.03	*0.07*
Excessive time	88.41	95.42	*0.06*
Anhedonia	56.94	79.25	<0.01^*^
Continuation despite complications	38.89	46.06	0.47
Total number of dependence criteria	4.75 (± 0.17)	5.53 (± 0.08)	<0.001^*^

* *Significant differences between groups according to independent samples t-tests or χ^2^ tests*.

The study of dependence criteria (DSM IV TR) for cocaine in the two groups showed significantly more withdrawal symptoms in the RDS+ group (*p* < 0.01). In this group (RDS+), there was also significantly more cessation of social or recreational activities that we define as anhedonia (*p* < 0.01). More persistent desire (*p* < 0.07) and excessive time spent around consumption (*p* < 0.06) were not significantly more frequent. Finally, the severity of dependence was significantly higher (*p* < 0.001) (RDS+ vs. RDS–: 5.53 ± 0.08 vs. 4.75 ± 0.17, respectively).

We included significant differences or clinically relevant variables in the FDA: the presence of psychotic symptoms, hallucinations, delusions, physical symptoms before use, and consumption-associated behavior (this group was named hyperdopaminergic component), and severity of dependence, presence of withdrawal symptoms, anhedonia and poly-drug use (this group was named hypodopaminergic component).

**Figure 1:**
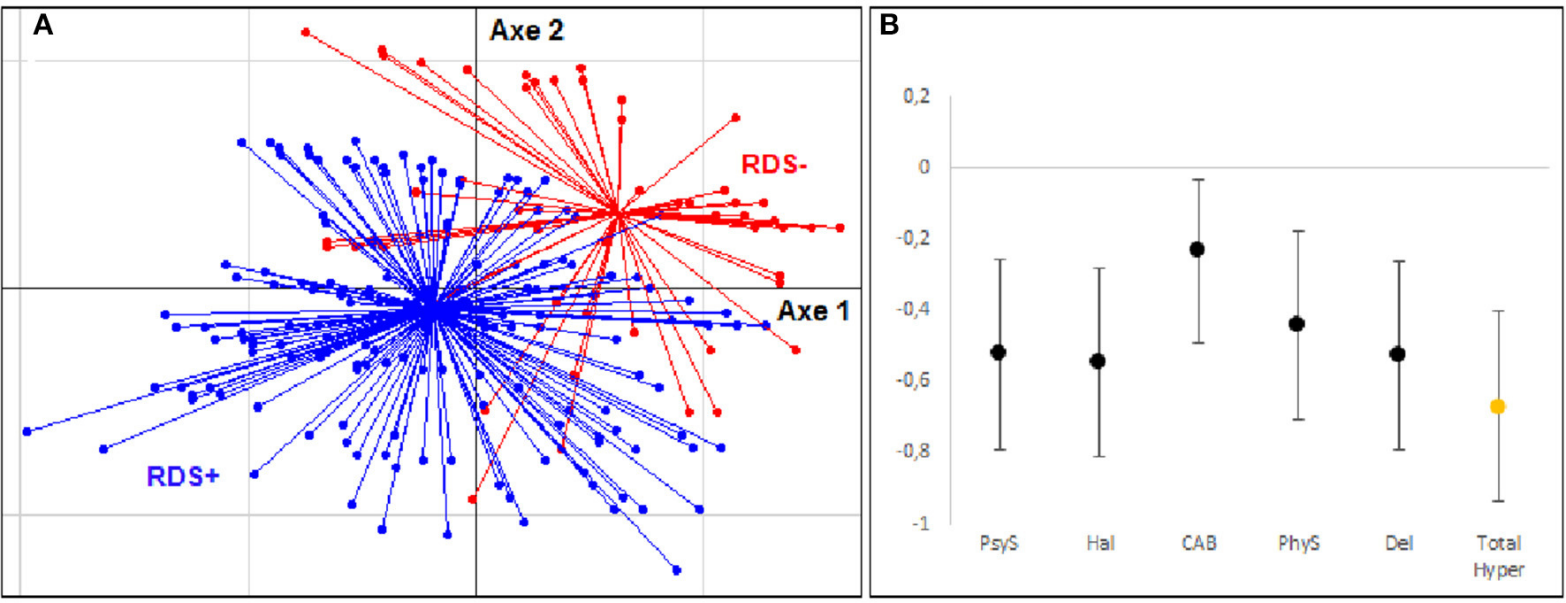
Multidimensional factor analysis and effect size for hyperdopaminergic components. **(A)** Tridimensional representation of the distribution of subjects with and without depressive symptoms during cocaine withdrawal (RDS– group in Red, RDS+ group in Blue) according to clinical variables of hyperdopaminergia: psychotic symptoms (PsyS), hallucinations (Hal), delusions (Del), consumption-associated behavior (CAB), physical symptoms before use (PhyS). **(B)** Hedges' effect-size of each variable for the hyperdopaminergic components.

The analysis (Figure 1A) revealed that in the hyperdopaminergic component, our population was well separated into two groups between RDS+ (blue) and RDS– (red) patients.

There were moderate to large effects size (Figure 1B) for psychotic symptoms (−0.524 ± 0.266) (PsyS), hallucinations (−0.548 ± 0.267) (Hal), and delusions (−0.528 ± 0.267) (Del). This effect appeared to be weaker for consumption-associated behavior (-0.231 ± 0.198) (CAB) and physical symptoms before use (−0.443 ± 0.266) (PhyS). The overall effect size of this component was large (−0.669 ± 0.269).

**Figure 2:**
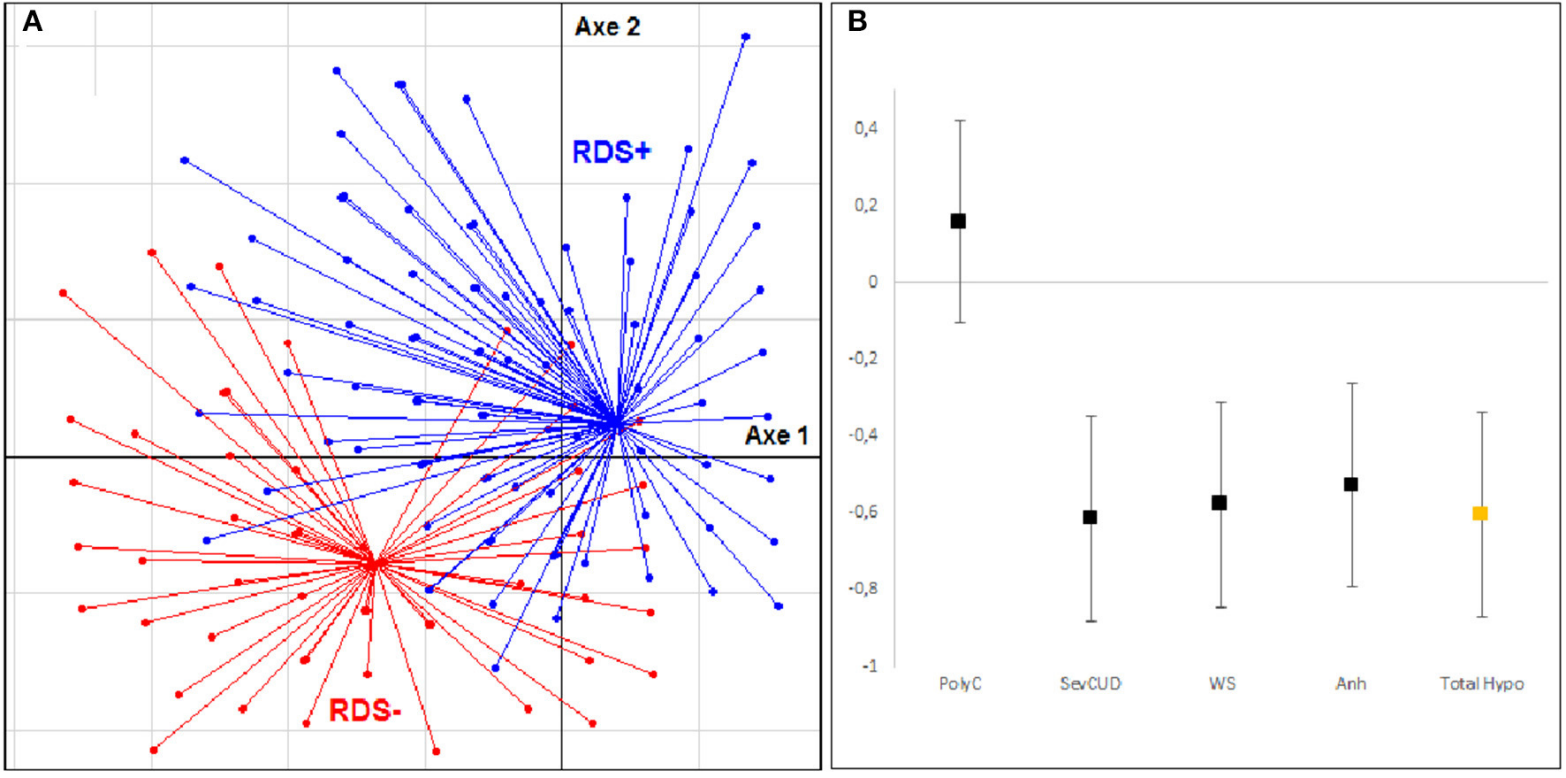
Multidimensional factor analysis and effect size for hypodopaminergic components. **(A)** Tridimensional representation of the distribution of subjects with and without depressive symptoms during cocaine withdrawal (RDS– group in Red, RDS+ group in Blue) according to clinical variables of hypodopaminergia: severity of dependence (SevCUD), withdrawal symptoms (WS), anhedonia (Anh), polyconsumption (PolyC). **(B)** Hedges' effect-size of each variable for the hypodopaminergic components.

For the hypodopaminergic component (Figures 2A,B), the analyses show a good separation of the RDS+ and RDS– groups. The effect sizes found were large for most of the variables: severity of dependence (−0.613 ± 0.264) (SevCUD), withdrawal symptoms (−0.578 ± 0.267) (WS), and anhedonia (−0.528 ± 0.266) (Anh). The effect size of the polyconsumption variable was small (0.158 ± 0.263) (PolyC). The effect size of the overall dimension was large (−0.604 ± 0.267).

**Figure 3:**
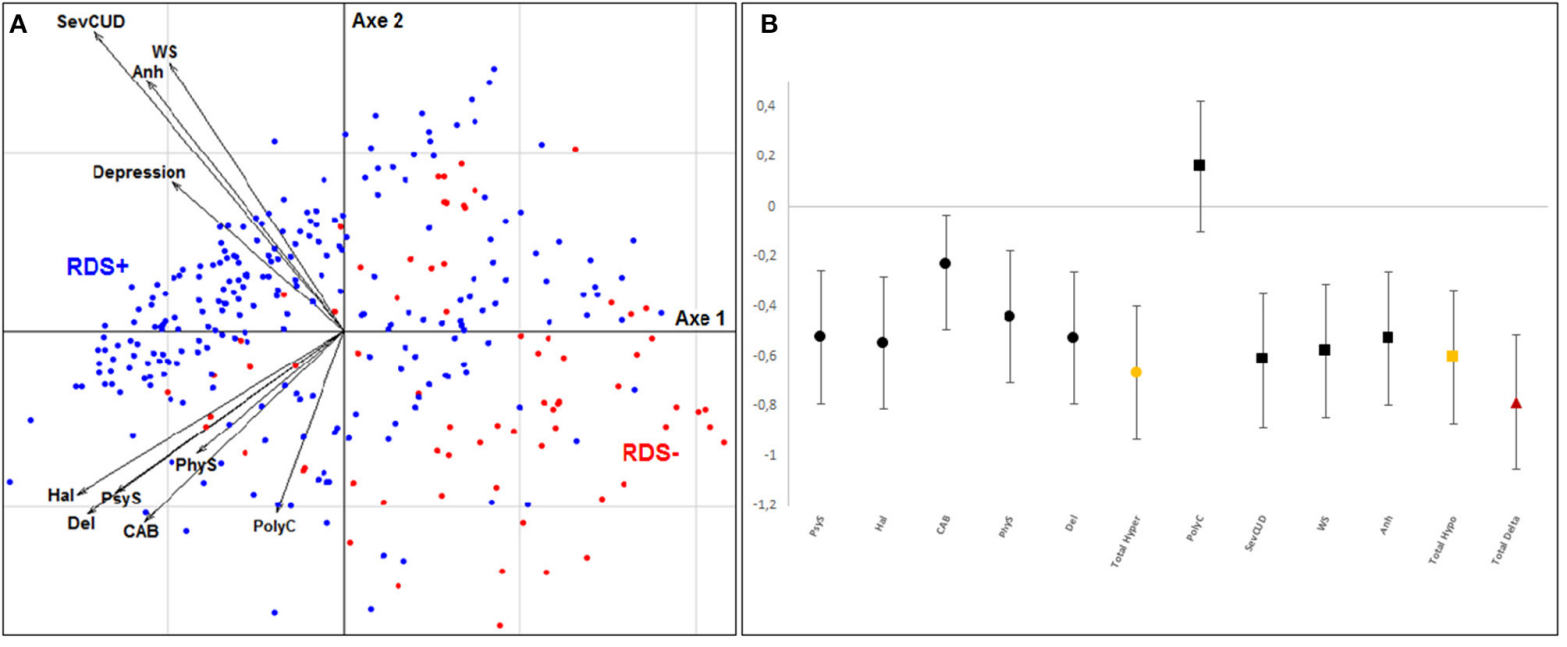
Multidimensional factor analysis and effect size for global model. **(A)** Tridimensional representation of the distribution of subjects with and without depressive symptoms during cocaine withdrawal (RDS– group in Red, RDS+ group in Blue) according to clinical variables of global dopaminergic model: severity of dependence (SevCUD), withdrawal symptoms (WS), anhedonia (Anh), polyconsumption (PolyC), psychotic symptoms (PsyS), hallucinations (Hal), delusions (Del), consumption-associated behavior (CAB), physical symptoms before use (PhyS); and direction of variables. **(B)** Hedges' effect-size of each variable for the global dopaminergic model components.

To evaluate our dopaminergic model in a global way, we carried out the same analysis by integrating all the variables. Figure 3A shows that the RDS + and RDS– subjects are again clearly separated into two groups. Figure 3A reveals on the left the existence of a group of subjects belonging to the RDS+ group and sharing very similar characteristics. On the right, the subjects belonging mainly to the other group are more dispersed. The overall effect size of our model was large (−0.785 ± 0.271).

This analysis finally allowed us to position the different variables in this model as vectors. Figure 3B shows the two main groups of variables that emerged: the first pointing upwards to the left and grouping variables of hypodopaminergic component, and the second pointing wards to the left and representing variables of hyperdopaminergic dimension. These two axes are well oriented toward the group of patients with depressive symptoms, which indicates that they help to explain this phenomenon.

## Discussion

This is one of the first studies to have evaluated mechanisms underlying the onset of depressive symptoms during cocaine withdrawal. The characteristics of our sample corresponded to those of European cocaine users (22, 27). Patients were predominantly single men, with an average age of onset of use of 22 years. They mainly used cocaine by inhalation, then by smoking and finally by injection. Most use was daily, and levels of polydrug use were high.

Our data showed that the RDS+ (Reported Depressive Symptoms +) and RDS– (Not Reported Depressive Symptoms) groups were homogeneous for age, gender, marital status, education, and history of hospitalization for withdrawal. The age of onset, frequency, and type of product most commonly used were also comparable in both groups. Levels of use of other psychoactive substances were identical in both groups, as was the level of polydrug use. These variables did not seem to have an influence on depressive symptoms, as reported by Uslaner et al. (28).

Concerning the modality of use, snorting was found more frequently in the RDS+ group, with depressive symptoms, (63.90 vs. 48.61%). Injectable or smoked pathways were more frequent in the RDS– group (26.39 vs. 23.65% for smoked; 18.06 vs. 7.86 % for injectable). This could be explained by the different pharmacokinetics of cocaine, depending on the form used. Clinical effects appear 3 mins after a cocaine snort and can last up to an hour. For the smoked route, they last only 10–30 mins, but are perceived in 5–10 s. The intravenous route acts in 16–20 s and has an effect lasting for 10–30 mins. In our depressed patients, snorting may therefore be a way to optimize intake so as to manage depressive symptoms more sustainably. This would be consistent, as the euphoric effects of cocaine are also perceived more intensely in depressed subjects compared to non-depressed subjects (29).

Depressive symptoms are more common among cocaine users than in the general population, with lifetime prevalence ranging from 25 to 61%, depending on the study (30, 31). Our patients have a higher rate of depressive symptoms (77%). Our recruitment sites could explain this phenomenon, as they were centers specialized in the management of opioid users. This corresponds to the data in the literature, which shows that depressive symptoms are found more particularly in patients who have entered the care process, probably because they are more symptomatic, and are associated with a more severe use disorder (31, 32). Our patients were therefore probably at a more severe stage than in other studies with higher recruitment. They had an average of 5.53 DSM IV dependence criteria. Bipolar disorders are also common in this population (33), and some authors confirm a predominance of depression among drug users (34).

The psychotic symptoms associated with cocaine use that we observed were mainly hallucinations, delusions, or stereotypies. They corresponded to those reported in the literature. These psychotic symptoms are found during consumption in 54–80% of patients (35, 36), and during dopaminergic treatments in Parkinson's disease (17, 37–39).

The choice of variables included in our analyses was crucial. We wanted to model the concepts of hyper and hypodopaminergy as well as possible by using indirect clinical markers of dopaminergic behaviors. Severity of dependence (*p* < 0.001), anhedonia (*p* < 0.01), and withdrawal symptoms (*p* < 0.01) were relevant for modeling clinical hypodopaminergic reactions in cocaine users. Psychotic symptoms and particularly hallucinations, delusions, and physical symptoms before use were criteria integrated into our modeling of clinical hyperdopaminergic disease. All of these criteria are consistent with those mentioned in several articles that have tried to clinically characterize these concepts in Parkinson's disease (PD), and define symptoms associated with hyperdopaminergia and hypodopaminergia. During the hyperdopaminergic phases in PD, certain researchers have observed delusions, hallucinations and motor stereotypes (17, 37, 38). More recently, hyperdopaminergia has also been linked to the development of behavioral addictions and still appears to be underestimated. They include compulsive purchases, pathological gambling and sexual behavior disorders (15). Symptoms associated with hypodopaminergia in PD are similar to depressive symptoms (15, 40), anxiety (41) and apathy (42).

Several studies have also investigated the release kinetics of dopamine in the nucleus accumbens during drug intake. In human subjects, a relationship between the subjective effects of cocaine and DA transporter (DAT) occupancy in the striatal areas has been demonstrated (13). Cerebral level curves for cocaine in the striatum are related to the cocaine-induced “high” behavior, with a peak at 10 mins then a progressive decrease of cerebral cocaine level and cocaine effects (13). These observations have also been found in animal models (43, 44). They show that the dopamine peak induced by stimulant intake in mice is almost immediate, followed by a progressive decrease over several tens of minutes or even hours.

Our patients were well discriminated in the analysis of the hyperdopaminergic component, and a large overall effect size of the hyperdopaminergic variables was observed. The most important criteria capable of explaining this difference between the groups were the existence of psychotic symptoms, particularly hallucinations and delirium, which is consistent with the scientific data (35, 36). Associated movements and physical symptoms before use seem to be involved but to a lesser extent. These Cocaine-Related Behavioral (CRB) symptoms are very frequently found in cocaine users, in particular for repetitive/stereotyped behaviors (45). The type of cocaine used does not influence these stereotypes (46), but on the other hand, these symptoms are found more frequently among cocaine injecting users, who were more numerous in the RDS– group. It therefore seems difficult to highlight a difference between our two groups on the basis of the sole criterion of depressive symptom. It would be interesting to study this phenomenon specifically in these injecting vs. non-injecting patients.

Patients were also well separated in the analysis of the hypodopaminergic component and there was a large overall effect size for these variables. Anhedonia, severity of dependence and symptoms appear to be good markers of this dimension. Our polyconsumption criterion, on the other hand, had only a small effect size. This could be explained by the low precision of the data for this variable, which is based on lifetime use of substances and not current or recent use. It might have been interesting to study comorbid opioid use disorder or Opioid Maintenance Treatment (OMT). A previous study using the same sample highlighted the role of OMT on the subjective effects produced by cocaine (23). Patients receiving an OMT at the time of their first cocaine use reported significantly less tachypsychia during this first cocaine intake, suggesting a specific protective effect of OMT on cocaine-induced “high” hyperdomapaminergic effects. Thus, a possible preventive effect on cocaine withdrawal would merit investigation.

These results show the interest of our two dimensions for exploring the phenomenon of depressive symptoms in cocaine users. When we integrated all these variables to perform a global analysis, discriminative capacity was even better, with a large overall effect size. This could indicate that rather than hypo or hyperdopaminergic changes occurring separately, these thymic variations might be explained by the switch from one to the other.

One of the main limitations of this study is that it was based on a cross-sectional study, and therefore causality cannot be established. It would have been interesting to perform a longitudinal evaluation of our two components. Even so, as we are interested in a short-term phenomenon, namely cocaine “comedown,” this cross-sectional analysis appears justified.

We chose to use a subjective assessment of depressive symptoms in cocaine users. This measure is more practical to use in this population, but it would have been appropriate to supplement this self-assessment with a more precise and detailed scale (24). Furthermore, using clinician-rated scales rather than self-reports could be useful if the tool is sufficiently time-sensitive.

Our sample may not perfectly represent the general population of cocaine users, but rather the most severe patients due to selection bias. Indeed, all of our patients were recruited from specialized addiction centers. The small number of subjects in our RDS- group (*N* = 72) is also a limitation.

In our overall model, we highlighted a homogeneous subgroup among RDS+ patients (see Figure 3A), but with a few scattered subjects. Other variables not available in our protocol (apathy and anhedonia) might play a role in this model to better condense the group, or to reveal subgroups that were not discriminated by our analyses.

In conclusion, our study was able to better characterize patients presenting depressive symptoms during cocaine withdrawal by comparing them with those who do not, according to their dopaminergic activity profile assessed by indirect clinical markers.

Early identification in the management process of these patients at risk of pejorative evolution or relapse could help to adapt the therapeutic strategy envisaged. The evaluation of depressive disorders in substance abuse patients might be helpful in designing and implementing specialized interventions to reduce the likelihood of relapse (7).

Further studies would be needed to study this phenomenon in more detail, with longitudinal evaluation and more accurate monitoring of symptoms. It would also be interesting to specifically study the very homogeneous group of RDS+ patients highlighted (Figure 3A). We could, for example, investigate physiological specificity by analyzing the genetic polymorphisms of the dopaminergic pathway.

## Data Availability Statement

The raw data supporting the conclusions of this article will be made available by the authors, without undue reservation.

## Ethics Statement

The studies involving human participants were reviewed and approved by Research Ethics Committee of Ile de France (Paris area). The patients/participants provided their written informed consent to participate in this study.

## Author Contributions

JC: conceptualization, methodology, data curation, writing—original draft preparation, writing—reviewing and editing, and visualization. GB: conceptualization, investigation, supervision, and writing—review and editing. BP: methodology, data curation, and formal analysis. NC and PL: conceptualization and writing—review and editing. EK, E-HZ, RI, and VB: conceptualization. FV: conceptualization, investigation, and writing—review and editing. ID: conceptualization, supervision, methodology, data curation, writing—review and editing, and formal analysis. All authors contributed to the article and approved the submitted version.

## Funding

The research project PSYCHOCOKE received a grant from the French Ministry of Health (Programme Hospitalier de Recherche Clinique National 2010, AOM10165), and was promoted by the DRCD (Direction de la Recherche Clinique et du Developpement) of the Assistance Publique–Hopitaux de Paris.

## Conflict of Interest

The authors declare that the research was conducted in the absence of any commercial or financial relationships that could be construed as a potential conflict of interest.

## Publisher's Note

All claims expressed in this article are solely those of the authors and do not necessarily represent those of their affiliated organizations, or those of the publisher, the editors and the reviewers. Any product that may be evaluated in this article, or claim that may be made by its manufacturer, is not guaranteed or endorsed by the publisher.

## Abbreviations

RDS+: patients who reported depressive symptoms during withdrawal
RDS–: patients who did not report depressive symptoms during withdrawal
PD: Parkinson's disease
OMT: opioid maintenance treatment.
